# Blood Selenium Concentrations Are Inversely Associated with the Risk of Undernutrition in Older Adults

**DOI:** 10.3390/nu15224750

**Published:** 2023-11-10

**Authors:** Esther García-Esquinas, Adrián Carballo-Casla, Rosario Ortolá, Mercedes Sotos-Prieto, Pablo Olmedo, Fernando Gil, Elena Plans-Beriso, Pablo Fernández-Navarro, Roberto Pastor-Barriuso, Fernando Rodríguez-Artalejo

**Affiliations:** 1 Department of Chronic Diseases, National Center for Epidemiology, Carlos III Institute of Health, 28029 Madrid, Spain; elena.plans@isciii.es (E.P.-B.); pfernandezn@isciii.es (P.F.-N.); rpastor@isciii.es (R.P.-B.); 2 Consortium for Biomedical Research in Epidemiology & Public Health (CIBER en Epidemiología y Salud Pública—CIBERESP), 28029 Madrid, Spain; adrian.carballo.casla@ki.se (A.C.-C.); rosario.ortola@uam.es (R.O.); mercedes.sotos@uam.es (M.S.-P.); fernando.artalejo@uam.es (F.R.-A.); 3 Aging Research Center, Department of Neurobiology, Care Sciences and Society Karolinska Institutet & Stockholm University, 141 86 Stockholm, Sweden; 4 Department of Preventive Medicine and Public Health, School of Medicine, Universidad Autónoma de Madrid, 28029 Madrid, Spain; 5 Department of Environmental Health, Harvard T.H. Chan School of Public Health, Boston, MA 02115, USA; 6 IMDEA Food Institute, CEI UAM+CSIC, 28049 Madrid, Spain; 7 Department of Legal Medicine, Toxicology, and Physical Anthropology, School of Medicine, University of Granada, 18071 Granada, Spain; polmedopalma@ugr.es (P.O.); fgil@ugr.es (F.G.)

**Keywords:** trace elements, protein-energy malnutrition, ageing, muscle weakness, body composition

## Abstract

Background: Selenium is an essential trace element with an antioxidant and anti-inflammatory capacity that has been associated in experimental studies with beneficial effects on appetite control, the regulation of the gut microbiota, and control of the anabolic–catabolic balance. The main aim of the present study was to evaluate the association between circulating selenium concentrations and the risk of developing undernutrition in older adults. Methods: This was a cohort study with 1398 well-nourished community-dwelling individuals aged ≥ 65 years residing in Spain in 2017, who were followed for a mean of 2.3 years. Whole blood selenium was measured at baseline using inductively coupled plasma-mass spectrometry. Undernutrition was assessed at baseline and at follow-up, and defined as having at least one of the three GLIM phenotypic criteria (involuntary weight loss, a low body mass index, and a reduced muscle mass) and at least one of the two etiologic criteria (reduced food consumption or nutrient assimilation and inflammation/disease burden). Results: During the follow-up, 142 participants (11%) developed moderate undernutrition and 113 (8.8%) severe undernutrition. The standardized relative risks of moderate and severe undernutrition at the 75th percentile of Se levels versus the 25th were 0.90 and 0.70, respectively. In dose–response analyses, the risk of severe undernutrition decreased linearly with increasing selenium concentrations. This association was independent of protein intake or diet quality and was stronger among participants with a diagnosis of a musculoskeletal disorder. Conclusions: The results suggest that an adequate dietary selenium status is needed to prevent undernutrition in older adults. Also, this may open the door for clinical trials with selenium supplementation, at doses considered as safe, to prevent undernutrition.

## 1. Background

Malnutrition is defined by the World Health Organization as a “deficiency or excess in nutrient intake, imbalance of essential nutrients or impaired nutrient utilization”. In older adults, protein–energy undernutrition is the most common form, with estimated prevalence rates in European populations ranging from 8.5% in community settings to 28% in hospitalized patients [1]. Protein–energy undernutrition results from a combination of a low macronutrient intake secondary to age-related changes in energy requirements, sensory functioning, and appetite, along with reduced assimilation, varying degrees of inflammation, and increased catabolism. This results in an altered body composition and decreased biological functions [2]. Protein–energy undernutrition is a major cause of adverse functional outcomes, such as sarcopenia or frailty, an increased frequency of hospital admissions, and all-cause mortality [3,4,5,6].

Selenium is a micronutrient naturally found in many foods such as grains, legumes, vegetables, nuts, fish and shellfish, dairy, or eggs. It plays a fundamental role in health, mainly due to the antioxidant, anti-inflammatory, and antiapoptotic capacity of selenoenzymes. In experimental models, several selenoproteins have demonstrated a modulatory effect on energy balance through the regulation of adipocyte activity, hypothalamic leptin signaling, orexin production, thyroid endocrine function, or insulin resistance [7,8,9,10]. The administration of selenium as seleno-DL-methionine to Se-deficient chicks increases voluntary feed consumption and body weight gain [11], while dietary supplementation with *p*-chloro-diphenyl diselenide in rats possesses anorexigenic effects through the modulation of food behavior, satiety signals, and diet palatability [12]. Also in rats, the administration of low-dose selenite during adolescence favors adipogenesis by promoting insulin secretion and sensitivity, leading to a general anabolism, while supplementation with selenium nanoparticles prevents fat depots and reduces inflammation in white adipose tissue [13]. On the other hand, the exposure of offspring dams rats to a restricted selenium diet during early programming promotes growth restriction, low insulin secretion, and the disruption of leptin and thyroid-stimulating hormone signals, as well as an extreme catabolic energy imbalance [14,15]. Moreover, combined aerobic interval training and selenium nanoparticle supplementation in mice prevents cachexia and muscle wasting in breast cancer bearers [16]. In another vein, selenium administration in mice influences both the intestinal gut microflora composition and the colonization of the gastrointestinal tract [17], and it alleviates intestinal barrier dysfunction by improving inflammation [18] and reducing oxidative stress [19], with a potential improvement in food absorption and assimilation.

Although severe selenium deficiency is rare in humans, decreased circulating levels in older adults have been associated with reduced circulating insulin-like growth factor (IGF-1) [20], reduced muscle mass [21] and strength [22], and increased mortality [23,24]. Additionally, the results of some randomized controlled trials in older adults have suggested positive effects of selenium supplementation on biomarkers of inflammation [25], oxidative stress [26], and insulin sensitivity [27]. Despite this evidence, and despite the fact that protein–energy undernutrition and micronutrient deficits frequently coexist [2], there are no epidemiological studies linking selenium status to undernutrition risk in community-dwelling older populations with an adequate intake. Here, we hypothesized that lower blood selenium levels are associated with undernutrition in old age. Accordingly, the main aim of the present study was to evaluate the association between circulating selenium concentrations and the risk of developing undernutrition in older adults.

## 2. Methods

### 2.1. Study Population and Design

The Seniors-ENRICA (Estudio de Nutrición y Riesgo Cardiovascular en España)-2 cohort (ClinicalTrials.gov NCT03541135) included 3273 community-dwelling adults aged ≥ 65 years, enrolled between 2015 and 2017 in the city of Madrid and four adjacent large cities (Getafe, Torrejón, Alcorcón and Alcalá de Henares). The participants were selected by stratified random sampling by sex and district from all national healthcare card holders (data available at: http://www.comunidad.madrid/servicios/salud/tarjeta-sanitaria, accessed on 13 September 2023). No exclusions were made on the basis of socioeconomic characteristics or medical conditions. Invitations to participate were sent through the post and with direct telephone contacts. The Clinical Research Ethics Committee of the “La Paz” University Hospital in Madrid approved the research protocol, and all participants gave written informed consent (Protocol #HULP-PI 1793) [28,29]. The study was performed in accordance with the World Medical Association’s Declaration of Helsinki for research involving human subjects.

Information from the participants was collected by trained personnel in different stages. First, a telephone interview was conducted on sociodemographic factors, health behaviors, self-rated health, and chronic morbidities. Next, during a home visit, trained nurses performed physical examinations (including grip strength, bioelectrical impedance, and anthropometric measurements), collected blood samples, and checked reported drug and supplement treatments with medication containers. Finally, in a further home visit, trained staff conducted an electronic and validated diet history with the participants. The median time between telephone interviews and physical examinations was 24 days, while the median time between the physical examinations and dietary histories was 7 days. Between 2018 and 2019, the participants were invited to update the study information, and the same data collection procedures were used as in the baseline wave. The mean follow-up time between baseline blood sample collection and undernutrition assessment was 27 months.

### 2.2. Study Variables

#### 2.2.1. Whole Blood Selenium

At baseline, fasting blood samples were collected in the morning and stored at −80°. Blood selenium was measured using inductively coupled plasma-mass spectrometry (8900 ICP-QQQ) at the Department of Legal Medicine, Toxicology, and Physical Anthropology, School of Medicine, University of Granada (Spain). No samples had levels below the limit of detection (0.3 µg/L). The inter-assay coefficient of variation (CV) for selenium levels was 5.24% [28]. Since circulating selenium shows a linear dose–response association with functional impairment, the Se concentrations were modeled as quintiles.

#### 2.2.2. Anthropometric Measures

Height measurements were conducted under standardized conditions using portable extendable stadiometers (Ka We 44 444 Seca), while weight and bioelectrical impedance were measured with electronic scales (Tanita^®^SC-240MA) [30]. Body mass index (BMI) was calculated as weight (in kg) divided by height (in meters) squared. Self-reported weight loss in the previous three and twelve months was also assessed [31]. Waist, mid-arm, and calf circumferences were measured by trained staff with a flexible, inelastic, belt-type tape [32]. Appendicular skeletal muscle mass was estimated from a predictive equation [4.957 + (0.196 ∗ height2/bioelectrical impedance) + (0.060 ∗ weight) − (2.554 ∗ sex)], as well as fat free mass [2.95 − (3.89 ∗ sex) + (0.514 ∗ height2/bioelectrical impedance) + (0.090 ∗ waist circumference) + (0.156 ∗ weight)] [33,34]. The appendicular skeletal muscle mass index and fat-free mass index were calculated as appendicular skeletal muscle mass and fat-free mass (in kg) divided by height (in meters) squared, respectively [35,36].

#### 2.2.3. Undernutrition

Undernutrition was assessed according to the Global Leadership Initiative on Malnutrition (GLIM), that is to say having at least one phenotypic (i.e., non-volitional weight loss, a low BMI, and reduced muscle mass) and one etiologic (i.e., reduced food intake or assimilation and inflammation/disease burden) criterion [35]. The phenotypic metrics for grading severity as Stage 1 (moderate) and Stage 2 (severe) malnutrition followed the GLIM guidelines [35], and so undernutrition was graded as moderate or severe if at least one phenotypic criterion met that grade of severity [35]. The operationalization of the undernutrition criteria was as follows: non-volitional weight loss was considered to be moderate when it was between 5 and 10% within the previous 3 months or 10–20% within the previous year, and severe if it was higher. A low BMI was considered to be moderate or severe if it was below 20 kg/m^2^ or 18.5 kg/m^2^ for those aged < 70 years, and below 22 kg/m^2^ or 20 kg/m^2^ for those aged ≥ 70 years, respectively. Muscle mass deficit was deemed moderate if grip strength, as assessed with a Jamar dynamometer [28], was <27 kg in men and <16 kg in women, and severe if, in addition to low strength, muscle mass was reduced (i.e., skeletal muscle mass of <20 kg in men or <15 kg in women, appendicular skeletal muscle mass index of <7 kg/m^2^ in men or <5.5 kg/m^2^ in women, fat-free mass index of <17 kg/m^2^ in men or <15 kg/m^2^ in women, mid-arm circumference of ≤21 cm, or calf circumference of <31 cm in both sexes).

Regarding the etiologic criteria, reduced food consumption or nutrient assimilation was defined as either an energy intake ≤50% of the requirements [37], a reduction in food consumption in the previous 3 months, or the presence of any chronic gastrointestinal condition, registered in the electronic health records, that may adversely impact nutrient assimilation or absorption [28]. Inflammation was deemed to impact nutritional status if either a selection of chronic diseases was present (i.e., chronic lung disease, heart failure, rheumatoid arthritis, liver disease, cancer, or chronic kidney disease) or supportive laboratory measures were altered (C-reactive protein of >3 mg/dL, serum albumin of <3.8 g/dL, or interleukin-6 levels of ≥7 pg/mL) [35,38,39].

#### 2.2.4. Dietary Intake

Habitual food consumption in the preceding year was assessed with a validated, computerized diet history that collected information on 880 foods and 34 alcoholic beverages, and used photographs to allow for classification in portion sizes [40]. The intake of energy, micro-, and macronutrients was estimated using standard food composition tables [41,42,43], and the recommended dietary allowance (RDA) was used as a reference to categorize the participants’ intakes of vitamins and minerals [44]. A minimum intake of 1.2 g of proteins per kg of weight/day was considered as adequate for the study population [45]. Adherence to the Mediterranean Diet was estimated using the Mediterranean Diet Adherence Screener (MEDAS) [46]. Standard beverage composition tables were used to estimate alcohol content, and the study participants were classified as non-drinkers, ex-drinkers, and current moderate or heavy drinkers (≥40 g/day for men and ≥24 g/day for women).

#### 2.2.5. Other Potential Confounders

During the interviews, information was collected on age, sex, and education attainment (<high school, high school, and >high school); living arrangements (alone, with a spouse/partner, and other); smoking (never, ex-smoker, and current smoker); recreational physical activity (Metabolic Equivalents of task-hours/week), estimated with the validated EPIC (European Prospective Investigation into Cancer and Nutrition)-cohort questionnaire [47]; television viewing time (hours/day), assessed with the Nurses’ Health Study questionnaire [48]; and history of physician-diagnosed chronic conditions. Cardiovascular disease (CVD) was defined as a diagnosis of coronary heart disease, congestive heart failure, heart attack, or angina. The definition of hypertension was based on a physician diagnosis, the current use of anti-hypertensive medication, or a blood pressure reading of 140/90 mmHg taken under standardized conditions. The definition of type 2 diabetes mellitus was based on a physician diagnosis, fasting glucose of ≥126 mg/dL, or the current use of anti-diabetic medication; and depression as having been diagnosed or requiring medical treatment.

### 2.3. Statistical Analyses

The selenium concentrations were markedly right-skewed, so they were log-transformed for the statistical analyses. The main characteristics of the participants according to their blood selenium quintile were described using either proportions (for categorial variables) or means and standard deviations (for continuous variables). Standardized relative risks (95% confidence interval) of undernutrition over follow-up according to the baseline whole blood selenium levels were estimated from multinomial logistic regression models, in which selenium concentrations were introduced as (1) quintiles; (2) 75th vs. 25th percentiles of log-transformed Se; and (3) cubic splines for log-transformed values with knots at the 5th, 35th, 65th, and 99th percentiles of the distribution. *p* values for a linear trend were obtained from Wald tests by introducing log-transformed selenium as a continuous variable. Also, departures from linearity in the restricted cubic spline models were evaluated using the Wald test.

Potential confounders were included in the models as follows: Model 1 was adjusted for age, sex, living arrangements (alone, with a spouse/partner, or other), and educational level (primary or less, secondary, or university); Model 2 was further adjusted for the MEDAS index, protein intake (g/day), and the number of vitamins with intakes above the RDA; and Model 3 was further adjusted for smoking (never, former, or current), alcohol consumption (never, former, moderate, or heavy drinker), recreational physical activity, sedentary behavior, and chronic morbidities (diabetes, cardiovascular disease, cancer, chronic lung disease, musculoskeletal disorders, and depression).

In sensitivity analyses, we tested for potential interactions between selenium concentrations and the participants’ sociodemographic, lifestyle-related, and clinical characteristics. Also, we further adjusted the models for biomarkers of inflammation (C-reactive protein, interleukin-6, and serum albumin), and for animal and vegetable protein intake.

All the analyses were performed using Stata 17.0 (StataCorp, Madrid, Spain).

## 3. Results

Of the initial sample of 3273 participants, 79.4% (2598) had whole blood selenium determinations available. Of these, we excluded the participants with prevalent undernutrition at baseline according to the Global Leadership Initiative on Malnutrition (GLIM) (*n* = 402), those who reported the intake of vitamins (Anatomical Therapeutic Chemical Classification System, code ATC11) or mineral supplements (ATC 12) (*n* = 135), and those with missing values of potentially important confounders (*n* = 69). Of the remaining 1992 participants, 28.9% were lost to follow-up and 0.9% died during follow-up, resulting in a final sample of 1398 older adults with updated information on undernutrition status.

At baseline, the median (interquartile range) concentration for whole blood selenium was 114.2 (102.8–126.5) µg/L. The mean age of participants was 71.0 years, 48% were women, and 21.6% had a university degree. Older participants, those with lower education, a lower adherence to the Mediterranean Diet and the RDA, or a lower protein intake, as well as those with diabetes or musculoskeletal disorders, had lower selenium concentrations (Table 1).

The reference group comprised participants with selenium concentrations below 99 µg/L.

**Figure 1:**
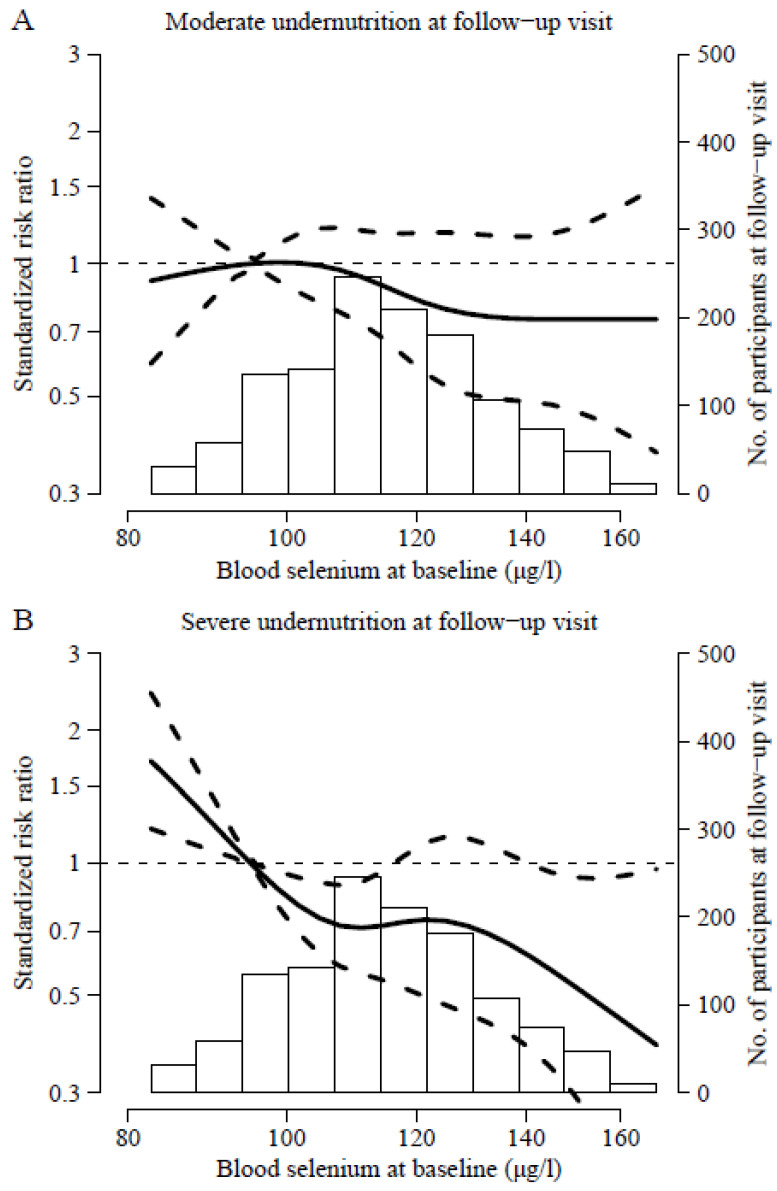
Standardized relative risk (95% confidence interval) of incident moderate (**A**) and severe (**B**) undernutrition according to baseline whole blood Se concentrations. Thick lines represent the standardized relative risks and dotted lines their 95% confidence intervals. Analyses are based on cubic splines for log-transformed Se with knots at the 5th, 35th, 65th, and 99th distribution. The reference value was set at the 10th Se percentile (95.0 ug/L). Relative risks were obtained from multinomial logistic regression models adjusted for age, sex, educational level, living arrangements, diet quality, protein intake, number of vitamins with intake above the recommended dietary allowance, smoking, alcohol consumption, recreational physical activity, TV-viewing time, and chronic morbidities (diabetes, cardiovascular disease, cancer, chronic lung disease, musculoskeletal disorders, and depression).

During the follow-up, 142 participants (11%) developed moderate undernutrition and 113 (8.8%) severe undernutrition. After multivariate adjustment, the standardized relative risks [95% confidence interval (CI)] of moderate and severe undernutrition comparing the 75th versus the 25th percentile of selenium concentrations were 0.90 and 0.70, respectively; the *p*-values for linear trend were 0.10 and 0.01, respectively (Table 2). When modeling the dose–response relationship for severe undernutrition using restricted cubic splines, we observed no significant deviations from linearity (*p*-nonlinearity = 0.25; Figure 1).

In the subgroup analyses, the standardized relative risks of severe undernutrition, comparing the 75th vs. 25th percentiles, were consistent across the participant characteristics except for the presence of musculoskeletal disorders at baseline (*p* for interaction = 0.039), with the strongest protective effects being observed among those with a prior diagnosis of osteoarthritis (0.56). Further adjustment for circulating levels of C-reactive protein, IL-6, and albumin yielded similar findings, with the relative risks (95% CI) of severe undernutrition comparing the 75th vs. 25th percentiles of selenium concentrations being 0.71, 0.71, and 0.69, respectively.

## 4. Discussion

In community-dwelling Spanish older adults, the selenium concentrations in the whole blood were inversely associated with the risk of severe undernutrition. This association was independent of protein intake or diet quality, and was shown to be stronger among participants with a previous diagnosis of a musculoskeletal disorder.

To our knowledge, this is the first study to evaluate the association between selenium exposure and the risk of undernutrition in community-dwelling older adults. However, previous studies have suggested an association between selenium deficiency and the risk of undernutrition in patients undergoing hemodialysis [49,50,51]. For instance, a cross-sectional analysis involving 118 middle-aged and older patients in maintenance hemodialysis found that lower blood selenium levels were associated with an elevated nutritional risk [50]. Also, a randomized double-blind placebo trial with 80 patients on hemodialysis treatment found that selenium supplementation for 12 weeks decreased the malnutrition-inflammation score and subjective global assessment score, both tools for assessing nutritional status, in the selenium group compared to placebo [51].

Undernutrition in community-dwelling older adults may be facilitated by a variety of age- and disease-related factors that have been associated with selenium deficiency and either promote reduced food intake and assimilation or lipolysis and loss of muscle mass. More specifically, depression, altered thyroid metabolism, and several cardiovascular and pulmonary diseases in the elderly often lead to unintentional weight loss and altered body composition through increased metabolic demand, decreased appetite, impaired nutrient absorption, and altered metabolism and excretion [52]. Through its influence in these conditions, selenium may thus promote undernutrition. For example, its deficiency has been associated with the prevalence of depression in older adults [53] and its genetically predicted concentrations have been linked to the risk of major depressive disorder in middle-aged adults [54,55]. Also, selenium has been involved in energy metabolism through the modulation of the IGF axis [20,27], insulin sensitivity [56], and leptin expression [54]. Indeed, a 4-year randomized controlled trial with 215 older individuals randomly assigned to supplementation with selenium and coenzyme Q10 or a placebo showed an increase in IGF-1 and postprandial IGF-binding protein-1 in the treatment group [27]. In a 3-month study of 37 overweight/obese individuals aged 18–65 years who adopted a mildly hypocaloric diet, those taking 240 μg/day of L-selenomethionine showed reduced leptin levels, a decreased fat mass, and an increased muscle mass compared to those on a placebo [54]. In relation to the apparent beneficial effect on muscle mass, some observational studies have also reported an inverse association between Se intake and the risk of sarcopenia in older adults [57,58], while selenium supplementation has been proposed as a promising strategy for reducing the risk of cachexia in cancer patients [59,60].

Among the potential confounders that could affect the validity of our results, we would like to emphasize inflammation and dietary quality. In this context, inflammation could mediate the redistribution of selenium from the plasma to the liver; hence, this results in reductions in blood concentrations that may not necessarily reflect true micronutrient deficiency [61]. However, adjustment for biomarkers of inflammation did not substantially alter the results. Additionally, considering that the some important sources of dietary selenium are protein-rich foods, and that poor dietary protein intake has been linked to undernutrition [62], a decrease in circulating selenium could indicate a reduced protein intake. Nevertheless, we observed only a modest correlation between protein intake and blood selenium concentrations (*p* = 0.12) and found no changes in the results when adjusting for animal and plant protein intake, or when conducting analyses stratified by tertiles of protein intake.

One of the limitations of the present study is the absence of repeated selenium measures, which would have allowed us to estimate the risk of selenium deficiency among individuals with malnutrition. Additionally, we did not measure selenoproteins, missing an opportunity to gain deeper insights into selenium’s mechanism of action concerning the risk of undernutrition. Regarding the outcome assessment, some biomarkers like serum albumin or prealbumin may have been influenced by unmeasured factors such as hydration status. Finally, given the transient nature of undernutrition, our 2.3-year follow-up might have overlooked short or mid-term changes in nutritional status.

## 5. Conclusions

Although the evidence is still insufficient, the results of the present work suggest that an adequate selenium status is needed to prevent undernutrition in older adults. Also, they may open the door for clinical trials assessing the effect of selenium supplementation at safe doses [63] on preventing undernutrition.

## Figures and Tables

**Table 1:** Distribution of participants’ characteristics according to quintiles of whole blood selenium (Seniors-Enrica 2, *n* = 1992).

Characteristics	Whole Blood Selenium (µg/L)				
	Q1	Q2	Q3	Q4	Q5
	(<99)	(99–110)	(110–120)	(120–130)	(≥130)
Number	367	382	400	420	423
Age (years), mean (SD)	73.06 (4.63)	71.89 (4.13)	71.37 (4.23)	71.18 (3.84)	70.79 (3.60)
Sex, %					
Men	47.68	53.93	52.75	50.48	47.99
Women	52.32	46.07	47.25	49.52	52.01
Education, %					
<High School	65.40	63.35	64.75	60.95	55.56
High School	20.16	18.32	18.00	21.67	19.15
>High School	14.44	18.32	17.25	17.38	25.30
Living arrangement, %					
Alone	74.66	77.49	78.25	80.24	75.89
With family members	23.16	21.73	20.00	18.33	23.17
Other	2.18	0.79	1.75	1.43	0.95
MEDAS, mean (SD)	6.74 (1.74)	6.96 (1.74)	7.13 (1.70)	7.48 (1.71)	7.39 (1.70)
Vitamins, above the RDA, %					
<5	45.78	41.10	40.50	36.67	29.31
≥5	54.22	58.90	59.50	63.33	70.69
Daily protein intake (g/kg), mean (SD)	1.21 (0.25)	1.24 (0.26)	1.24 (0.23)	1.27 (0.24)	1.30 (0.26)
Physical activity METs-hours/week, mean (SD)	65.24 (36.76)	68.50 (37.75)	68.60 (32.71)	68.97 (33.84)	69.25 (37.34)
Watching TV h/week, mean (SD)	24.06 (11.67)	21.69 (11.34)	22.17 (10.46)	21.61 (10.41)	21.08 (9.71)
Smoking, %					
Never	48.50	50.26	47.50	52.86	50.12
Former	40.33	37.43	41.75	39.29	42.32
Current	11.17	12.30	10.75	7.86	7.57
Alcohol consumption, %					
Never	22.62	17.28	14.75	16.43	15.84
Former	66.76	73.82	75.00	72.86	71.39
Moderate drinker	5.72	4.19	4.25	6.43	6.86
Heavy drinker	4.90	4.71	6.00	4.29	5.91
Hypertension, %	69.48	70.68	67.75	67.14	66.43
Cardiovascular disease, %	4.36	2.09	2.50	1.67	2.60
Diabetes mellitus, %	24.25	21.99	20.00	20.71	14.18
Cancer, %	3.00	2.36	2.00	1.67	3.07
Osteomuscular disease, %	40.33	33.25	29.50	35.24	33.33
Depression, %	6.27	4.97	5.75	3.81	3.07

MEDAS: Mediterranean Diet Adherence Screener; METs; Metabolic equivalent of tasks; RDA: Recommended Dietary Allowance; SD: Standard Deviation; Q: Quartiles and TV: Television.

**Table 2:** Standardized relative risk (95% confidence interval) of undernutrition risk, according to baseline whole blood Se levels.

	Whole Blood Selenium ( )						
	Quintile 1	Quintile 2	Quintile 3	Quintile 4	Quintile 5	*p*-trend	Per Interquartile Range
	(<99)	(99–110)	(110–120)	(120–130)	(≥130)		
GLIM definition							
Moderate undernutrition	30	32	30	29	21		142
Model 1	1.00	1.06 (0.66–1.70)	0.97 (0.60–1.57)	0.91 (0.56–1.47)	0.67 (0.39–1.14)	0.06	0.86 (0.71–1.05)
Model 2	1.00	1.07 (0.67–1.71)	1.00 (0.62–1.62)	0.94 (0.57–1.53)	0.70 (0.41–1.21)	0.08	0.88 (0.72–1.07)
Model 3	1.00	1.07 (0.67–1.72)	1.04 (0.64–1.67)	0.98 (0.60–1.60)	0.71 (0.41–1.23)	0.10	0.90 (0.74–1.10)
Severe undernutrition	33	22	15	21	22		113
Model 1	1.00	0.65 (0.39.1.09)	0.43 (0.24–0.77)	0.58 (0.34–0.98)	0.60 (0.36–1.01)	0.04	0.77 (0.61–0.96)
Model 2	1.00	0.62 (0.38–1.02)	0.40 (0.22–0.70)	0.53 (0.31–0.89)	0.49 (0.29–0.82)	0.01	0.70 (0.55–0.88)
Model 3	1.00	0.64 (0.39–1.06)	0.41 (0.24–0.73)	0.55 (0.33–0.91)	0.51 (0.31–0.87)	0.01	0.71 (0.57–0.89)

Model 1: Adjusted for sex, age, living arrangements (alone, with a spouse/partner, or other) and educational level (primary or less, secondary, or university). Model 2: As Model 1 and additionally adjusted for diet quality (MEDAS index), protein intake (g/kg/day), and number of vitamins with intake above the recommended dietary allowance. Model 3: As Model 2 and additionally adjusted for smoking (never, former, or current), alcohol consumption (never, former, moderate, heavy drinker), recreational physical activity, TV-viewing time, and chronic morbidities (diabetes, cardiovascular disease, cancer, chronic lung disease, musculoskeletal disorders, and depression).

## Data Availability

The data presented in this study are openly available in https://repisalud.isciii.es/.
